# Comparisons Between Adolescent Bullies, Victims, and Bully-Victims on Perceived Popularity, Social Impact, and Social Preference

**DOI:** 10.3389/fpsyt.2019.00868

**Published:** 2019-11-22

**Authors:** Alexa Guy, Kirsty Lee, Dieter Wolke

**Affiliations:** ^1^Department of Psychology, Staffordshire University, Stoke-On-Trent, United Kingdom; ^2^Department of Psychology, University of Warwick, Coventry, United Kingdom; ^3^Department of Psychology, University of Ottawa, Ottawa, ON, Canada; ^4^Warwick Medical School, University of Warwick, Coventry, United Kingdom

**Keywords:** bullying, victimization, peer status, peer relationships, adolescence

## Abstract

This study investigated the effect of bullying role, i.e., bully, victim, and bully-victim, on three measures of peer status; perceived popularity, social preference, and social impact. In addition to completing peer nominations for these measures of peer status, adolescents (n = 2,721) aged 11 to 16 years from 5 secondary schools completed an online survey that assessed bullying involvement (self- and peer-reported), self-esteem, and behavioral difficulties. Compared to uninvolved adolescents, all bullying roles had a greater social impact. Bullies scored higher than all other roles for perceived popularity, whereas victims and bully-victims were the lowest in social preference. These significant group comparisons remained when controlling for demographic variables, behavioral difficulties, self-esteem and prosocial behavior. Overall, the perceived popularity found for bullies suggests that these adolescents are socially rewarded by peers for their victimization of others. These findings highlight the need to address the whole peer system in raising the social status of those who are victimized, whilst reducing the rewards received by bullies for their behavior.

## Introduction

School bullying is a highly pervasive issue for children and adolescents world-wide, yet despite extensive efforts to identify the motivations behind bullying and ways to tackle it, interventions have been mixed in their success (1). Resource control theories propose that some aggression may be functional and can lead to potentially adaptive outcomes (2, 3) and, for some adolescents, bullying may be an effective form of aggression that is used to gain or maintain social dominance (4, 5). However other adolescents who bully are reported to be socially marginalized and rejected by their peers (6, 7). This has led to the identification of two subgroups of perpetrators: bullies and bully-victims (i.e. those who bully others but are also victimized). Bully-victims are often impulsive, high in reactive aggression, and have been reported to have poor social skills; including biases in social information processing (8, 9). Conversely, bullies are considered to be proactive and strategic in their use of aggression, and have a competent social cognition (10, 11). The differences between bullies and bully-victims in their social and behavioral characteristics may influence their status amongst the peer group in different ways. Exploring the status profiles of these perpetration groups, compared to purely victimized or uninvolved adolescents, could highlight potential social motivations behind bullying behavior.

An individual's social standing within the peer group can be represented by two similar yet distinct constructs: social preference and perceived popularity (12). Social preference represents how accepted or 'liked' a person is (1, 13), and is typically measured by asking participants to nominate peers whom they most and least like, or most and least want to hang around with (14). Perceived popularity on the other hand reflects an individual's social prestige and dominance within the peer group, and is most commonly measured from peer-nominations of who are the most popular and least popular members of the classroom (5). Although these two aspects of peer status are often moderately correlated (15), they are distinct constructs; those who are popular are not always accepted by peers. Social preference is commonly associated with positive social attributes, such as cooperativeness (16), whereas perceived popularity may be influenced by characteristics such as attractiveness, athleticism, or having desirable possessions (17, 18). Social impact is a third aspect of peer status that refers to the prominence or visibility of an individual within the peer group (19), and has been used to determine status hierarchies in classrooms (19, 20). Thus, social impact is a measure of how visible or known a student is within the social group (e.g. classroom) however, although an individual with high social impact may have a high social presence, their overall status profiles can either be positive or negative, or indeed both.

Aggression has shown associations with perceived popularity, whereby aggressive youth are often reported to be popular, despite being largely disliked by others (13, 21). Similarly, some bullies have been found to be highly popular, but often have lower social preference than their uninvolved peers (22, 23). Low social preference however has not always been found for adolescent bullies (17), and this has led to reports that many bullies have controversial status within the peer group; i.e., they are liked by some and disliked by others (24, 25). Victims on the other hand have been reported to be low in both perceived popularity and social preference (5, 22), and may therefore be easy targets for bullies (26). Similarly peers may avoid being affiliated with victims through fear of jeopardizing their own status or being targeted themselves (27, 24). Bully-victims have been reported to be the most ostracized by peers (6, 28, 29), and therefore their bullying of others may be ineffective in achieving the same perceived popularity as the 'pure' bullies. Studies involving child and/or pre-adolescent samples have reported that bully-victims are overall less accepted and more rejected than bullies (29–31), yet despite their distinct behavioral and psychological profiles, bully-victims are not consistently assessed independently from bullies and victims (32, 33).

In addition to their involvement in bullying, adolescent bullies, victims, and bully-victims possess distinct attributes that could be either valued or considered undesirable by the peer group. Bullies are reported to be confident, have high self-esteem, and are often perceived as 'cool' amongst their peers (34, 35), while victims may often lack self-esteem (36) and show difficulties with emotion regulation (37, 38). Bully-victims are reported to be highly reactive and have been associated with the worst emotional and behavioural difficulties (39). These attributes may influence an adolescent's status amongst their peers, and therefore it is unclear whether bullying role specifically has an effect on peer status, over and above these individual characteristics.

Two widely employed methods to measure bullying involvement are self-reports and peer-nominations (40, 41). These methods typically produce different prevalence estimates of bullying and victimization, and specifically how many are identified as bullies, victims, or bully-victims. There is a risk of bias within self-reports, whereby individuals may not admit to bullying others, or have biased perceptions of their behavior. Self-report measures commonly result in an under-reporting of bullying perpetration; approximately 1–5% (28, 42), whereas peer-reports often yield higher rates of 13–14% (29,). Although peer-nominations reduce the risk of subjective errors, they ultimately rely on how much of the bullying or victimization is visible to the peer group (44). In particular, victimization may often not be visible to the peer group and sometimes hidden. Therefore, a combination of self- and peer-reports may be necessary for investigating differences between the groups involved in bullying, whilst retaining sufficient statistical power.

The primary aim of this study was to investigate differences between adolescent bullies, victims, bully-victims, and those not involved (using a combination of self- and peer-reports) on three measures of peer status: social impact, social preference, and perceived popularity. Secondly, the effect of bullying role on these status measures, when controlling for other individual (e.g. emotional and behavioral problems, self-esteem) and demographic factors (e.g. gender, age) was assessed. In line with previous findings, despite much of this literature pertaining to younger children (45), it was predicted that adolescent bullies would be highest in perceived popularity but lower in social preference than those not involved. Victims were hypothesized to be lower in perceived popularity than bullies and to have lower social preference than uninvolved adolescents. It is not clear how bully-victims would compare to other roles with regards to perceived popularity, yet they were expected to be lower in social preference than those not involved in bullying. Finally, all those involved in bullying were expected to have higher social impact than uninvolved adolescents; although it is unclear whether social impact would vary between bullies, victims, and bully-victims.

## Materials and Method

### Design and Sample

**Figure 1:**
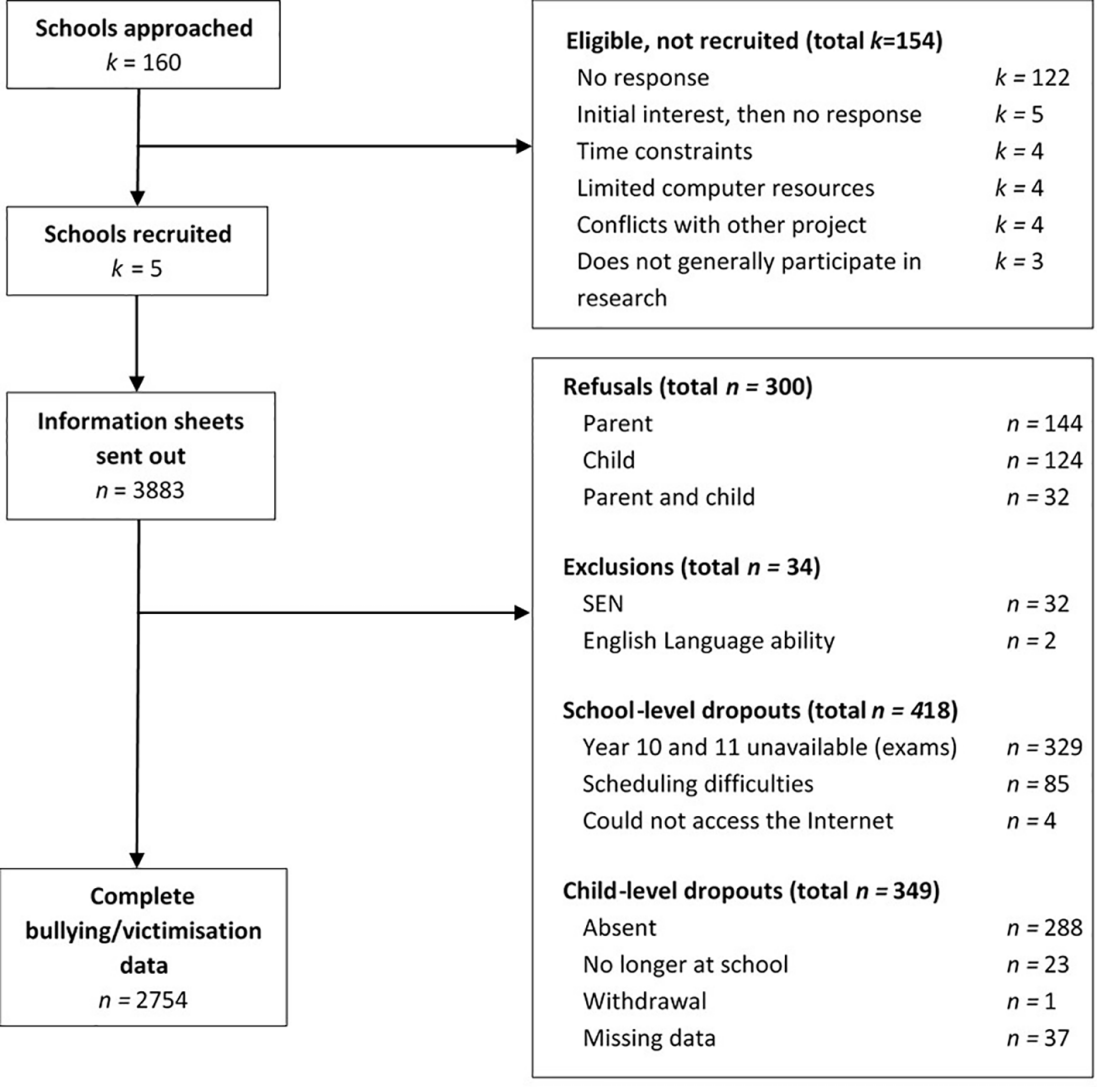
Flow diagram of recruitment and selection of schools and participants.

Data was collected during stage one of the BASE Study (Bullying, Appearance, Social Information Processing and Emotion Study; 36, 46, 47); a two-phased study that assessed a range of physical, social, and emotional attributes in relation to bullying involvement in adolescence. Pupils aged 11–16 years (*N* = 3,883) from five secondary schools in Central England, United Kingdom, were recruited into the study. Schools were mostly mixed-faith, mixed-gender (except for one girls' grammar school), and represented different social-economic backgrounds. Following child and/or parent refusals, dropouts (i.e., non-participation due to, for example, pupil absence or school scheduling difficulties), and exclusions (see **Figure 1**), the final sample comprised 2,754 pupils with complete data for the bullying/victimization items (female; 56.8%, White British; 82.6%, age in years; *M* = 13.51, *SD* = 1.35).

All participants gave their informed consent and full ethical approval for the study was obtained from the University of Warwick's Ethics Committee.

### Procedure

Schools were contacted and sent written details about the study. Once a school's involvement was confirmed, all pupils (aged 11–16 years) and their parents received information sheets and consent forms. Pupils could only participate if they had provided signed consent, and their parents had not returned a refusal form for their child's participation. The online assessment was completed in groups of 20–30 pupils (approximately 50 min) during the school day. At the start of each session, pupils were provided with a written overview about the study, and were given standardized instructions for completing the assessment. The survey was accessed *via* individual passwords, and could only be completed when at least one researcher and teacher were present.

### Measures

#### Bullying Involvement

For self-reported bullying/victimization, the bullying and friendship interview schedule (48) was used. First, pupils were given 13 behavioral descriptions of victimization (36); five items related to direct victimization (e.g., “been called nasty names”), four items to relational victimization (e.g., “been made to do things you didn't want to do”), and four items related to cyber-victimization (e.g., “had rumors spread about you online”). Pupils were asked how often they had experienced each behavior in the last six months; never, sometimes, quite a lot (several times a month), or a lot (at least once a week). The same items were adapted to assess bullying perpetration. Self-reported victims were pupils who responded with “quite a lot” or “a lot” to any of the 13 victimization items; self-reported bullies were pupils who responded with "quite a lot" or "a lot" to any one of 13 bullying items; and bully-victims were those pupils who had been identified as both a self-reported victim and bully (49, 50). Good reliability was found for the victimization (α = .84) and bullying (α = .86) items.

For peer-nominated bullying involvement, pupils were given a numbered list of students in their tutor/form group (broadly equivalent to the 'homeroom' in US schools). Participants were asked to nominate up to three pupils (including non-participating pupils), by selecting their corresponding number on screen, who were either victims or perpetrators of the behaviors described (e.g., for relational bullying; “Some people repeatedly leave people out of get-togethers, parties, trips or groups, get others to ignore people, or spread nasty lies, rumors, or stories about people on purpose. Which people in your form/tutor do this?”). To account for the variable number of 'nominating' participants in each tutor group, the victimized and bullying nominations were standardized within tutor groups to create a 'bullying' and 'victimization' z-score for each participant. Pupils were identified as a peer-nominated bully if their z-score was greater than one for the bullying items, and peer-nominated victims were those with z-scores >1 for the victimization. Finally, pupils with z-scores >1 for both the victimization and bullying items were classified as peer-nominated bully-victims. This study limited nominations to three pupils to encourage participants to consider who best fits the descriptions, rather than simply nominating most classmates (5, 31).

#### Peer Status

Social impact, social preference, and perceived popularity were assessed using a standard peer-nomination procedure (5, 20, 51). For social impact and social preference, pupils were asked to nominate up to three members of their tutor group who they most and least wanted to hang around with. Participants could not nominate themselves, and could respond with “Nobody,” “I don't know,” or “I don't want to answer.” Peer-nominations were summed and standardized within tutor groups to create separate z-scores for the 'most liked' and 'least liked' nominations. Social impact was calculated by summing the most and least liked z-scores, and a social preference score was obtained by subtracting the least liked z-score from the most liked z-score (20).

Similarly, for perceived popularity, participants were asked to nominate up to three classmates who were the 'most popular' and 'least popular'. Perceived popularity was then calculated by subtracting the standardized 'most popular' z-score by the 'least popular' z-score (5).

#### Behavioral and Emotional Difficulties

The strengths and difficulties questionnaire (SDQ) (52) has been widely used to assess behavioral and emotional difficulties, and prosocial behavior in 11–17 year-olds (53). This self-report measure consists of 25 items grouped into five subscales: hyperactivity, emotional symptoms, peer problems, conduct problems, and prosocial behavior.

Participants responded on a three-point scale; from 0 = not true to 2 = certainly true, to indicate how much they agreed with each statement. A score for each subscale was calculated by summing responses from the corresponding items. Higher scores indicate more difficulties, except for the prosocial subscale in which higher scores reflect more prosocial behavior. The peer problems subscale addresses aspects of peer victimization and popularity, and was therefore excluded from the analyses. Additionally, one item was removed from the conduct problems subscale as it described behavior associated with bullying. A total difficulties score was obtained by summing the hyperactivity, emotional symptoms and conduct problems subscales, with higher scores indicating more difficulties. Cronbach's alpha for total difficulties was.71 and.70 for the prosocial behaviour subscale.

#### Self-Esteem

Participants completed Rosenberg's Self-Esteem (SE) Scale (54), which includes 10 self-report items, with responses on a four-point scale; from 0 = “disagree a lot” to 3 = “agree a lot.” All items were reverse-coded, whereby higher scores indicated lower self-esteem, and responses were summed to create a total self-esteem score. Cronbach alpha for the current sample was α = .89.

#### Individual Characteristics

Pupils reported their gender, ethnicity, date of birth, and their parent's highest level of education (i.e., 1–11 years; no education to basic schooling, and >11 years; further education, college or university). Ethnicity was dichotomized into 'White British' and 'Other' due to the low prevalence of individual ethnic groups (e.g., 'Asian' was the next largest group at 6.1%). Schools provided data regarding participants' attendance (%) and pupil premium status. In the UK, pupil premium refers to extra funding that schools receive for disadvantaged pupils (including pupils who have been eligible for free school meals in the past six years). Pupil premium status for each participant ('yes'/'no') was obtained as an indicator of deprivation and/or financial assistance.

### Analysis

Participants with whole scales missing for the self-reported bullying and victimization measure were excluded from the final sample, along with participants with more than one missing item per scale. Missing data for a single item was replaced with the mean value for that scale (stratified by gender), and bivariate analyses found no significant differences in bullying role or any demographic variable between those with complete or missing data.

Analysis of variance (ANOVAs) or chi-square comparisons were conducted to compare scores between the bullying roles for each of the demographic variables, and participants' scores for self-esteem, total difficulties (calculated from the difficulties subscales of the strength and difficulties questionnaire; SDQ) and prosocial behavior (the prosocial subscale of the SDQ). ANOVAs and Bonferroni adjusted post-hoc comparisons were also conducted to identify differences in the mean scores for social impact, social preference, and perceived popularity between the bullying roles. Finally, analysis of covariance (ANCOVAs) and Bonferroni adjusted post-hoc comparisons were conducted to compare mean scores between the bullying roles for social impact, social preference, and perceived popularity, whilst controlling for all demographic variables, scores for self-esteem, total difficulties and prosocial behavior (which were entered as covariates).

Eta squared (*η* ^2^) and partial eta squared (*ηρ* ^2^) is reported as a measure of effect size; with values of.0099,.0588, and.1379 as indicators of small, moderate, and large effect sizes, respectively (55, 56). Statistical significance was set at *p* < .05 and all analyses were computed using SPSS version 22.

## Results

### Final Sample

Thirty-Three Pupils Were Identified as Missing From the Tutor Group Lists or Included on the Incorrect List. These Pupils Could Therefore Not Be Nominated by Other Participants and Were Excluded From the Analyses. the Final Sample of Participants Was Therefore 2,721; Female = 56.9%; White British = 82.4%; Age in Years; M = 13.51, SD = 1.36 (**Table 1**).

**Table 1:** Descriptive data for final sample (split by bullying role). All numbers are percentages, unless otherwise stated.

	N (%)	Total	Bully	Victim	Bully-victim	Uninvolved	Differences between bullying roles
		2721	279 (10.3)	649 (23.9)	390 (14.3)	1403 (51.6)	
Gender	*Female %*	56.9	49.5	58.7	46.7	60.3	*χ* *^2^* = 14.68, ***p*** ** = .002**
	*Male %*	43.1	50.5	41.3	53.3	39.7	
Age (years)	*Mean*	13.51	13.88	13.36	13.73	13.44	*F*(3,2717) = 11.87, ***p*** ** < .001**
	*(SD)*	(1.36)	(1.38)	(1.34)	(1.29)	(1.36)	
Ethnicity	*White British %*	82.4	80.7	82.2	82.7	82.8	*χ* *^2^*(3) = 1.17, ***p*** = .760
	*Other %*	17.6	19.3	17.8	17.3	17.2	
Attendance	*Mean*	95.60	95.07	95.11	95.15	96.07	*F*(3,2263) = 9.08, ***p*** ** < .001**
	*(SD)*	4.64	4.52	5.40	4.78	4.17	
Parent Ed	*≤11 years %*	12.3	13.3	13.3	14.6	11.0	*χ* *^2^*(3) = 5.92 *p* = .116
	*> 11 years %*	87.7	86.7	86.7	85.4	89.0	
PP	*No %*	78.1	71.2	73.7	70.2	83.7	*χ* *^2^*(3) = 46.49, ***p*** ** < .001**
	*Yes %*	21.9	28.8	26.3	29.8	16.3	
SDQ	*Mean*		12.87	14.77	16.63	10.67	*F*(3,2664) = 130.41, ***p*** ** < .001**
	*(SD)*		(5.91)	(6.49)	(6.55)	(5.68)	
Prosocial	*Mean*		11.61	12.10	11.13	12.10	*F*(3,2717) = 16.98, ***p*** ** < .001**
	*(SD)*		(2.54)	(2.26)	(2.96)	(2.53)	
SE	*Mean*		20.48	23.17	23.28	20.25	*F*(3,2717) = 57.54, ***p*** ** < .001**
	*(SD)*		(5.86)	(6.12)	(6.66)	(5.08)	
Impact	*Mean*		.276	.056	.292	-.154	*F*(3,2717) = 19.35, ***p*** ** < .001**
	*(SD)*		(1.244)	(1.257)	(1.360)	(1.173)	
Preference	*Mean*		-.050	-.190	-.513	.239	*F*(3,2717) = 31.68, ***p*** ** < .001**
	*(SD)*		(1.411)	(1.537)	(1.812)	(1.344)	
Popularity	*Mean*		.691	-.369	-.077	.079	*F*(3,2717) = 31.499, ***p*** ** < .001**
	*(SD)*		(1.553)	(1.611)	(1.854)	(1.376)	

Parent Ed, parent's education; PP, pupil premium status; SDQ, total difficulties; SE, self-esteem.

### Bullying Roles

For self-reported bullying involvement, the percentage of participants identified in each of the four bullying groups were; bullies 2.2%, victims 21.7%, bully-victims 6.5%, and uninvolved 69.6%. For peer-nominated bullying involvement, the percentage of participants identified within each group were; bullies 13.2%, victims 12.1%, bully-victims 5.2%, and uninvolved 69.5%. Pupils were then assigned to a final 'combined' bullying role (see **Table 1**), based on the scores obtained for both the self-reported and peer-nomination measures (36). Bullies were either a self-reported or peer-nominated bully (and not also a self-reported or peer-nominated victim), and victims were those identified as either a self-reported or peer-nominated victim (and not also a self-reported or peer-nominated bully). For the combined bully-victim role, participants were; 1) either a self-reported bully-victim or peer-nominated bully-victim, 2) either a self-reported victim and a peer-nominated bully, or 3) a self-reported bully and a peer-nominated victim. Participants who were not identified as a bully, victim, or bully-victim on the self-report or peer-nomination measures were categorized as uninvolved.

### Differences Between Bullying Roles for Demographic Variables and Scores for Self-Esteem, Total Difficulties, and Prosocial Behavior

Demographic data for each bullying group is reported in **Table 1**, in addition to the mean scores for total difficulties, prosocial behavior, self-esteem, and each of the peer status variables (social impact, social preference, and perceived popularity). The results of the Bonferroni adjusted group comparisons (chi-squares, one-way ANOVAs) are also displayed.

Of the demographic variables, gender (*χ* *^2^*(3) = 14.68, p = .002), age (*F*(3,2717) = 11.87, *p* < .001), attendance (*F*(3,2263) = 9.08, *p* < .001), and pupil premium status (*χ* ^2^(3) = 46.49, *p* < .001) showed significant differences between the bullying roles (**Table 1**). There were significantly more males identified as bully-victims than victims (*p* = .002) and those uninvolved (*p* < .001), and the perpetration groups (bullies and bully-victims) had a higher mean age than both the uninvolved and victim group (*p* < .001). Uninvolved adolescents had significantly higher attendance at school than bullies (*p* = .027), victims (*p* = .001), and bully-victims (*p* < .001), and there were significantly less uninvolved adolescents with pupil premium status compared to the other groups (*p* < .001).

There was also a significant main effect of bullying role on total difficulties (*F*(3,2664) = 130.41, *p* < .001), self-esteem (*F*(3,2717) = 57.54, *p* < .001), and prosocial behavior (*F*(3,2717) = 16.98, *p* < .001). For total difficulties, there were significant differences between all of the groups (all *p* < .001), whereby those uninvolved had the lowest scores, followed by bullies, and bully-victims overall showed the highest levels of difficulties. Bullies and uninvolved adolescents had significantly higher self-esteem than both victims and bully-victims (*p* < .001). Bullies (*p* = .032) and bully-victims (*p* < .001) had lower levels of prosocial behavior than the uninvolved group, and victims were significantly higher in prosocial behavior than bully-victims (*p* < .001)

### Differences Between Bullying Roles for Peer Status

**Figure 2:**
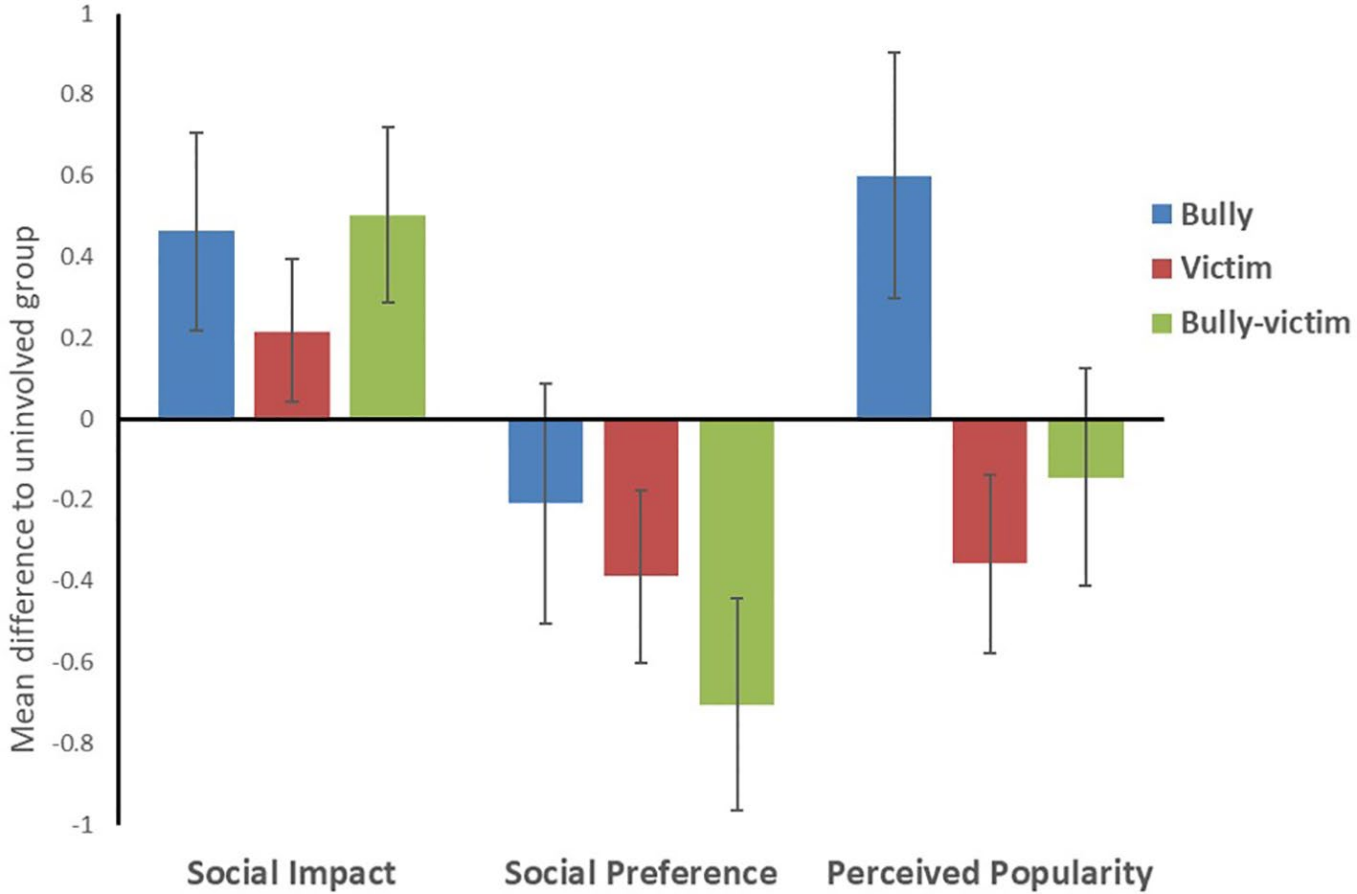
Mean differences in social impact, social acceptance, and perceived popularity between bullying roles (bullies, victims, bully-victims) and the uninvolved group (represented at the zero line).

One-way ANOVAs were first conducted to compare bullying roles on social impact, social preference, and perceived popularity (**Table 1**). All demographic variables, and scores for total difficulties, self-esteem, and prosocial behavior that showed significant differences between the groups, were then entered as covariates into the model. Adjusted means for and Bonferroni comparisons (whilst controlling for gender, age, attendance, pupil premium status, total difficulties, self-esteem, and prosocial behavior), are reported for the bullying roles in **Table 2**. Finally, **Figure 2** shows the mean differences in z-scores between the 'involved' roles (bullies, victims, and bully-victims) and those not involved for social impact, social preference, and perceived popularity.

**Table 2:** Adjusted means and comparisons between bullying roles (Bonferroni adjusted) for social impact, social preference, and perceived popularity.

		Social Impact^1^			Social Preference^2^			Perceived Popularity^3^		
		M	SE	95% CI	M	SE	95% CI	M	SE	95% CI
Role	Uninvolved	-.170 ^a^	.038	-.243, -.096	.224 ^a^	.045	.135, .313	.054 ^a^	.046	-.037, .145
	Bully	.292 ^b^ ^c^	.085	.126, .458	.015 ^a^ ^b^	.102	-.186, .215	.653 ^b^	.105	.447, .858
	Victim	.047 ^b^	.054	-.059, .152	-.166 ^b^	.065	-.293, -.039	-.304 ^c^	.066	-.434, -.174
	Bully-victim	.333 ^c^	.070	.195, .471	-.481 ^c^	.085	-.647, -.314	-.090 ^a^ ^c^	.087	-.260, .081

Role means are adjusted for the inclusion of covariates: gender, age (in years), attendance, pupil premium status and scores for self-esteem, total difficulties and prosocial behavior.

For each model, roles that do not share the same superscript (^a b c^) are significantly different at the p < .05 level.

^1^Significant covariate(s); prosocial behavior only (F(1,2197) = 5.72, p = .017).

^2^Significant covariate(s); age (F(1,2197) = 6.75, p = .009), attendance (F(1,2197) = 9.56, p = .002), pupil premium status (F(1,2197) = 4.52, p = .034), and self-esteem (F(1,2197) = 4.33, p = .037).

^3^Significant covariate(s); age (F(1,2197) = 30.48, p < .001), self-esteem (F(1,2197) = 12.89, p =. < 001), and total difficulties (F(1,2197) = 5.76, p = .016).

#### Social Impact

In the unadjusted model, bullying role had a significant main effect on social impact (*F*(3,2717) = 19.35, *p* < .001, *η²* = .021), whereby the uninvolved group were significantly lower in social impact than bullies (*p* < .001), victims (*p* = .002), and bully-victims (*p* < .001). Moreover, bully-victims were significantly higher in social impact than victims (*p* = .017). When adjusted for the inclusion of covariates, the significant main effect of bullying role remained (*F*(3,2197) = 17.25, *p* < .001, *ηρ²* = .023), whereby the uninvolved group were lower in social impact than bullies (*p* < .001), victims (*p* = .007), and bully-victims (*p* < .001). Bully-victims also remained significantly higher in social impact than victims (*p* = .006).

#### Social Preference

The one-way ANOVA for social preference found a significant main effect of bullying role (*F*(3,2717) = 31.68, *p* < .001, *η²* = .034). Bully-victims had significantly lower social preference than bullies (*p* = .001), victims (*p* = .004) and the uninvolved group (*p* < 001), and victims were also significantly lower in social preference compared to those uninvolved (*p* < .001). With the inclusion of covariates in the model, the main effect of bullying role remained significant (*F*(3,2197) = 19.18, *p* < .001, *ηρ²* = .026), whereby bully-victims were significantly lower in social preference than bullies (*p* = .001), victims (*p* = .004), and the uninvolved group (*p* < 001). Uninvolved adolescents also remained significantly higher in social preference than victims (*p* < .001).

#### Perceived Popularity

In the unadjusted model for perceived popularity, there was a significant main effect of bullying role (*F*(3,2717) = 31.50 *p* < .001, *η²* = .034); whereby bullies had significantly higher levels of perceived popularity than all other groups (*p* < .001). Moreover, victims were significantly lower in perceived popularity than bully-victims (*p* = .019) and those uninvolved (*p* < .001). When adjusted for the inclusion of the covariates, the significant main effect was maintained (*F*(3,2717) = 31.50 *p* < .001, *ηρ²* = .027); with bullies remaining higher in perceived popularity than all other groups (*p* < .001), and victims scoring significant lower than those uninvolved (*p* < .001). The difference between victims and bully-victims however was no longer significant.

## Discussion

The primary aim of this study was to explore the peer status of adolescents involved in bullying by making direct comparisons between those involved (i.e., bullies, victims, and bully-victims) and those uninvolved on social impact, social preference, and perceived popularity. Secondly, the influence that involvement in bullying has on peer status, above other demographic and individual characteristics, was investigated. Bullying role had a significant main effect on all aspects of peer status. Compared to uninvolved adolescents, all those involved in bullying had higher social impact. In comparison to all other roles, bullies had higher levels of perceived popularity, whereas bully-victims were lower in social preference. These differences remained when controlling for demographic variables, and scores for overall difficulties, self-esteem, and prosocial behavior.

These findings support previous claims that during adolescence, bullies often have a dominant position within the peer group (22, 23). In this study, bullies had higher perceived popularity than their victimized and uninvolved peers, and although it is uncertain if their perceived popularity is a direct result of bullying others, this suggests that bullies incur few social costs from their behavior (57). Moreover, bullies were not significantly lower in social preference than those uninvolved, which supports previous findings that bullies in fact have an average level of social preference (17), and overall a controversial status amongst peers (24, 58, 59).

With regards to resource control theories of aggression, bullying may be used to access resources or gain social dominance (32, 60), and for many bullies, this behavior may be successful in achieving high social status (4). Thus, the high levels of perceived popularity associated with this group could act as both a motivation and a reward for their behavior (23, 32). It is possible however that this group may possess other characteristics that contribute to their popular status (18); i.e., they may be strong, athletic, or physically attractive. Bullies have also been described as callous, strategic, and manipulative (32, 61) and therefore able to adopt more sophisticated and hidden forms of bullying (62), or coax peers into believing that the bullying is justified (26). These traits and characteristics, along with a reputation for rule-breaking that many peers see as 'cool' (23, 35), may help bullies maintain their dominant status within the peer group (63).

Conversely, victims have been associated with characteristics that may make them vulnerable for victimization and its persistence over time; i.e., being anxious, sensitive, or lacking confidence (26, 64, 65). In the adjusted model, victims were perceived as less popular compared to non-victimized adolescents (i.e., bullies and uninvolved), and this low popularity can be considered both a consequence of being bullied and a risk factor for victimization (5). Bullies may see those with lower social status as 'easy targets', and believe there is less risk of being punished by the peer group for selecting these targets (45, 66). Victims in this study also had lower social preference than uninvolved peers. Victims may avoid social situations (67), but also peers may be reluctant to be affiliated with a known victim through fear of jeopardising their own social position or becoming targets themselves (24). Positive peer relationships are reported to provide resilience against victimization (68, 69), and therefore the attitudes of the whole peer group should be addressed to provide more social support and ultimately raise the social status of victimized youth.

Bullies and bully-victims both had high levels of social impact, however they were different across the other measures of peer status. Bully-victims were significantly lower in social preference and perceived popularity than bullies, and this may reflect potential differences in the way that aggression is used between these two groups. Bully-victims may represent the coercive and socially marginalized aggressors described by resource control theories (7). These adolescents may lack efficient cognitive, social, and emotional skills (8, 38, 39), and fail to successfully use a combination of coercive and prosocial strategies in their pursuit of social dominance (2, 70). Bully-victims could therefore experience feelings of hopelessness and social defeat (71), and this could account for some of the adverse physical and psychological outcomes reported for this group (39, 72, 73). Thus, although adolescent bullies and bully-victims appear to have a similar impact on their social worlds, their social experiences are distinct (74), and our findings show that having high social impact is not necessarily a positive attribute for overall peer status.

There are a number of limitations to this study. Firstly, the design was cross-sectional and therefore causality cannot be inferred from the associations reported. Longitudinal studies have reported a bi-directional association (17, 34), and some suggest that bullying/victimization and popularity reinforce each other over time (34, 45). Secondly, the findings relate to pupils from the five secondary schools recruited, and although these schools showed socioeconomic and ethnic diversity, they may not be representative of the UK as a whole, although prevalence and patterns match those of a recent nation-wide study (75). Thirdly, a number of potentially influential physical characteristics (i.e., attractiveness or athleticism) were not assessed. These attributes have shown associations with both popularity and bullying/victimization (23, 76), and have been reported to strengthen the relationship between bullying and popularity (77). It is, therefore, possible that having positive physical attributes, along with other peer-valued characteristics, could influence the associations reported here, and have potentially varying effects on status outcomes for males and females (18).

A major strength of this study was the combined use of self- and peer-reports to identify those involved in bullying. A low agreement between informants has been shown in research in other areas such as mental health (78); where the use of multiple informants and combining measures is recommended for more accurate assessment of pervasive mental health problems (78–80). This low agreement has also been shown previously for bullying roles between self-report and peer-nominations, with only a small number of bullies are identified by self-reports (81, 82). Studies using self-reports have reduced statistical power to systematically investigate bullies, even in large samples (83). In this study, we combined the self- and peer-reports, which reduced the risk of shared variance with the peer status measures, whilst retaining the statistical power of the comparisons. Researchers should work towards reaching a consensus in how bullying and victimization is measured in order to produce more consistent and comparable findings across studies.

In conclusion, adolescent bullies, victims, and bully-victims have a greater impact on their social worlds than those not involved in bullying. Bullies receive social rewards in the form of increased perceived popularity amongst peers, whereas those who are victimized appear to be neither the popular nor accepted members of the classroom. Changing the behavior of a popular bully is a challenging task, and thus, alternative and ultimately more prosocial means by which bullies can maintain their social status should be promoted within child and adolescent populations (1). The contribution that being a bully, victim, or bully-victim has on peer status, suggests a need to address the whole peer group in order to improve the social status of victims and bully-victims, and inhibit the social environment that allows bullies to thrive.

## Data Availability Statement

The datasets generated for this study are available on request to the corresponding author.

## Ethics Statement

This study was carried out in accordance with the recommendations of BPS ethical guidelines and the University of Warwick's ethics committee, with written informed consent from all subjects. All subjects gave written informed consent in accordance with the Declaration of Helsinki. The protocol was approved by the University of Warwick's ethics committee.

## Author Contributions

AG and DW conceived the study and all authors contributed to the study design. AG and KL carried out the data collection. AG produced the first draft of the manuscript and all authors revised the manuscript and approved the final version.

## Funding

AG and KL were supported to undertake this research by a fellowship from the Department of Psychology, University of Warwick.

## Conflict of Interest

The authors declare that the research was conducted in the absence of any commercial or financial relationships that could be construed as a potential conflict of interest.
